# Transient Dyschromatopsia, Static Form Agnosia, and Prosopagnosia Observed in a Patient with Anti-NMDA Receptor Encephalitis

**DOI:** 10.1155/2019/2929782

**Published:** 2019-04-02

**Authors:** Ritsuo Hashimoto, Asako Tagawa, Noriyo Komori, Tomoko Ogawa, Hiroyuki Kato

**Affiliations:** ^1^Department of Neurology, International University of Health and Welfare Hospital, Japan; ^2^Department of Neurology, Hiratsuka City Hospital, Japan; ^3^Department of Language and Hearing Sciences, International University of Health and Welfare, Japan

## Abstract

We presented a case of a 19-year-old woman who suffered from anti-*N*-methyl-D-aspartate (NMDA) receptor encephalitis associated with ovarian teratoma. The patient showed a variety of higher visual symptoms which changed over the recovery phase of the disease. In chronological order, she experienced cortical blindness, amblyopia, dyschromatopsia, static form agnosia, and prosopagnosia. Among these symptoms, the most intriguing was the static form agnosia. Although she could recognize the forms of moving objects, she could not make out those of stationary ones. All of these visual symptoms were transient, implying that she might have incidentally regained the function of the distinct cortical visual areas in the time course of follow-up. This case further suggests that visual functions concerned with the perceptions of static form and form-from-motion could be dissociable and may rely on distinct brain regions.

## 1. Introduction

Anti-*N-*methyl-D-aspartate (NMDA) receptor encephalitis is an autoimmune disease first reported over a decade ago among women who were diagnosed with ovarian teratoma and who presented with a variety of neurological symptoms, including behavioral changes, hallucinations, memory deficits, seizures, decreased level of consciousness, and central hypoventilation [1]. A definitive association between anti-NMDA autoantibodies and encephalitis was established by Josep Dalmau and colleagues in 2007 [2]. Here, we report a case of a young woman with anti-NMDA receptor encephalitis associated with ovarian teratoma who showed a variety of cortical visual symptoms including dyschromatopsia, static form agnosia, and prosopagnosia during the recovery phase of the disease. Among these symptoms, the most intriguing was the static form agnosia, wherein she could recognize the forms of moving objects but not of stationary objects. We discuss a possible underlying mechanism of the symptom.

## 2. Case Report

**Figure 1:**
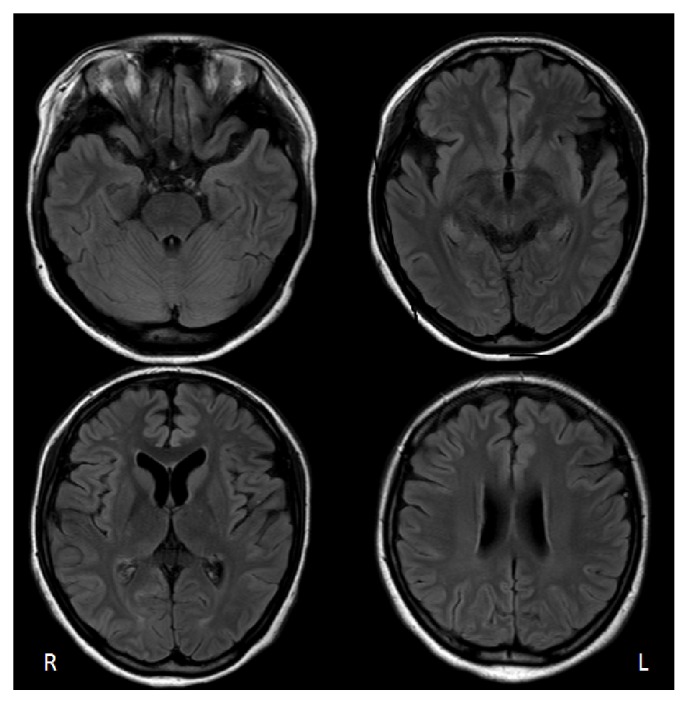
Axial fluid-attenuated inversion recovery (FLAIR) images of the brain two months after admission. Note the slight high intensities in the bilateral occipital cortices and subcortical white matter, which were somewhat prominent on the right side, as well as small subcortical high intensities in the right frontal lobe. However, there were no signal abnormalities in the medial temporal areas.

A 19-year-old right handed woman was referred to our hospital with complaints of persistent fever and headache from 3 days before. At the time of initial symptoms, her family members reported strange behavior as the patient had repeatedly asked the same questions. On admission, she still retained consciousness and could recognize her family members, but was disorientated to time and place. Magnetic resonance imaging (MRI) on admission demonstrated no signal abnormalities in the brain, including the medial temporal areas. Cerebrospinal fluid (CSF) examination on admission revealed lymphocyte dominant pleocytosis (lymphocytes 168/mm^3^, polymorphonuclear cells 13/mm^3^) with a protein level of 46 mg/dL (normal 15–40 mg/dL) and a sugar level of 61 mg/dL (normal 50–70 mg/dL). A clinical diagnosis of encephalitis was made and an intravenous administration of acyclovir, fosphenytoin sodium hydrate, and glycerol was initiated. On the second day of admission, she developed repeated generalized tonic-clonic convulsions leading to a convulsive status. She required supportive therapy involving intubation, mechanical ventilation, and sedation. A test for herpes simplex virus (HSV)-PCR of the CSF was negative. Antibodies against GluN1/GluN2 heteromers of the NMDA receptor were detected in serum at two different times (Table 1). From this evidence, a diagnosis of anti-NMDA receptor encephalitis was made. Two months after admission, fluid-attenuated inversion recovery (FLAIR) images of brain MRI showed areas of slightly high intensity in the bilateral occipital cortices and subcortical white matter, which were somewhat prominent on the right side, as well as small subcortical high intensities in the right frontal lobe (Figure 1). Over the following several months, orofacial dyskinesia and bibrachial spasm occurred frequently despite immunotherapy with high-dose methylprednisolone (1000 mg/day for 3 days) twice and intravenous immunoglobulin (400 mg/kg/day for 5 days) once. She was weaned off the ventilator 6 months after admission, though she was still unconscious.

**Figure 2:**
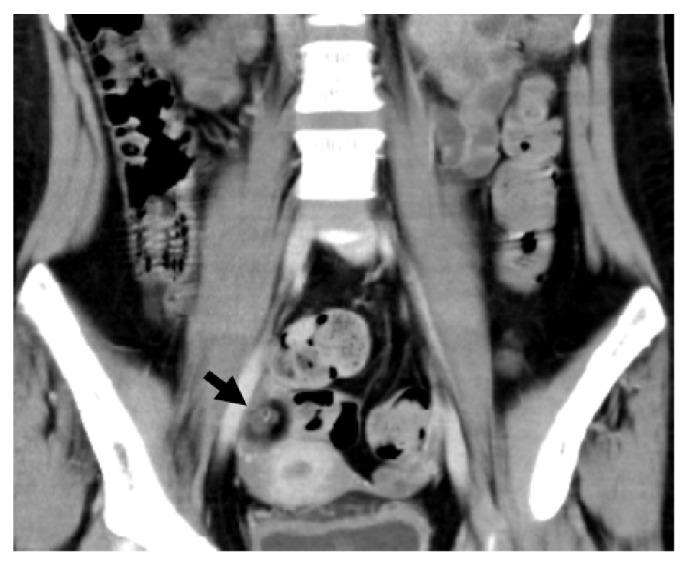
Abdominal CT after 8 months of admission demonstrating a small ovarian calcified cystic mass on the right side (arrow).

Abdominal computed tomography (CT) and MRI for the purpose of detecting ovarian tumor were both negative on initial examinations. However, a repeated abdominal CT after 8 months of admission revealed a small ovarian calcified cystic mass on the right side (Figure 2). The patient had the tumor removed, which was diagnosed as a mature cystic ovarian tumor on pathological examination. After the removal of the tumor, she received high-dose methylprednisolone and intravenous immunoglobulin therapy again. Three months after these treatments (12 months after admission), the patient's consciousness improved gradually and she was slowly able to respond to verbal commands.

At this time, she stated that she could not see anything. Ophthalmological examination revealed that pupillary reaction and extraocular movements were normal. In addition, there were no abnormalities in the anterior and posterior components of the eyes, including the lens, retina, and optic fundi. The patient's exact visual acuity was difficult to estimate because she complained of fatigue and often refused to continue the test. Regarding color perception, she could differentiate a red cup from a blue cup; however, she could not distinguish a blue cup from a green cup. On behavioral observations, she behaved like a blind person; although she could walk while holding hands with a caregiver, she frequently collided with doors. When she attempted to pick up her comb or toothbrush from a table, she explored these objects by palpating with her hand across the table. She could not recognize stationary objects by visual inspection alone, but she could identify them by tactility or hearing the sounds that they made. Around this time, it was incidentally noticed that she could successfully respond to the examiner's motions. For example, when the examiner waved their hand, she waved back. On a different occasion, when the examiner swayed their body, the patient immediately imitated the movements. These observations suggested the patient was able to see moving objects better than stationary objects, despite her serious visual impairment.

**Figure 3:**
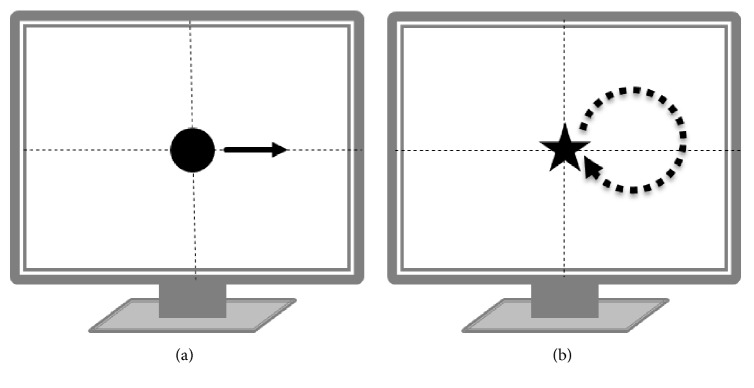
(a) Dot detection task. The patient was tested to see if motion of a visual stimulus affected her visual ability. A small black dot 0.4 cm in diameter was presented in the center of the screen for 3 seconds with no motion (stationary condition) or with right side lateral motion with a velocity of 2 cm/sec (moving condition). (b) Figure recognition task. The patient was tested to see if motion of a figure could influence her ability to identify it. Four different figures black in color were prepared; a circle, a star, a triangle, and a square, each of which was inscribed in a circle with a diameter of 1 cm. Each figure was presented in the center of the screen with no motion for 4 seconds (stationary condition) or with clockwise rotatory movement from the center tracing on a circle of 4 cm diameter with a velocity of 2 cm/sec for 4 seconds (moving condition).

Subsequently, we further examined her visual abilities in detail. First, the patient was asked to detect stationary visual stimuli presented on a computer screen placed 30 cm from her eyes. We prepared two different stimuli, a black dot with 1 cm in diameter (approximately 2 degrees in visual angle) and another with 0.4 cm in diameter. They were presented randomly for three seconds on each of the five points, comprised of four corners and the center of the screen. She was able to identify the larger stimuli perfectly (10/10: correct/total) indicating that she had no obvious hemianopsia or quadrantanopsia of the cortical type, although she often missed the smaller ones (2/10: correct/total). Second, the patient was tested for her ability to detect the motion of a visual stimulus. A small black dot 0.4 cm in diameter was presented in the center of the screen for 3 seconds with no motion (stationary condition), or with rightward lateral motion with a velocity of 2 cm/sec (moving condition) (Figure 3(a)). She was able to detect the moving stimuli better (8/10: correct/total) than stationary ones (1/10: correct/total). Third, the patient was tested to see if the motion of a figure influenced her ability to identify the shape of the figure. Four different figures in black: a circle, a star, a triangle, and a square, were inscribed in a circle with a diameter of 1 cm. Each figure was presented in the center of the screen with no motion for 4 seconds (stationary condition) or with a clockwise rotational movement from the center tracing on a circle of a 4 cm diameter with a velocity of 2 cm/sec for 4 seconds (moving condition) (Figure 3(b)). She showed a tendency to recognize the shape of moving figures better (7/18: correct/total) than stationary ones (3/18: correct/total) (Table 2). The test results suggested that the patient retained some capacity of form-from-motion perception: i.e., an ability to recognize the shape of an object under motion.

The patient's visual symptoms and general cognitive function gradually improved. Fifteen months after admission, she was able to recognize stationary objects as well as moving ones in everyday life. She also was able to pick up an object with appropriate preshaping of her fingers. At this time, the patient scored 28/30 on the revised Hasegawa dementia scale (normal >21/30). Further, we administered Visual Perception Test for Agnosia (VPTA) to her. On VPTA, a mistake or failure in performance is each scored 1 or 2 points according to the criteria. Thus, greater score indicates more severe visual disability. The patient showed a partial impairment in her basic visual perception (VPTA score 6/24), yet she was able to read kana and kanji characters. Notably, we observed severe impairment in perceiving famous faces (VPTA score 10/10). She could not tell if a photograph of a person was young or old, or man or a woman, but was able to identify if two faces presented were identical or not. The patient also demonstrated difficulties in identifying family members by visual inspection alone, stating “I can recognize my family members when I hear their voice but cannot by sight alone because their faces look dark and ambiguous.” On the other hand, her color perception had recovered to near normal levels (VPTA score 2/30).

She was discharged 18 months after admission, and had become able to identify family members and medical staff on sight, with no complaints of any visual symptoms. Brain MRI performed on discharge showed no signal abnormalities except for subcortical small high intensities in the occipital lobes.

## 3. Discussion

NMDA receptor encephalitis involves antibodies against the GluN1 subunit of the NMDA receptor. These antibodies probably cause the binding, capping, and cross-linking of NMDA receptors leading to internalization of the receptors from the neuronal membrane surface, resulting in dysfunction of signal transmission mediated by glutaminergic synapses [3, 4]. A previous study of brain metabolism patterns using fluorodeoxyglucose (FDG)-PET/CT revealed that decreased occipital lobe metabolism was more prominent in patients with anti-NMDA receptor encephalitis compared to other definite autoimmune encephalitis [5]. Furthermore, the study reported that among patients with anti-NMDA receptor encephalitis, patients with the greatest neurologic disability were more hypometabolic compared with less severe disability in the visual cortical and occipital brain regions [5]. Thus, it is not surprising that patients with anti-NMDA receptor encephalitis, especially those with serious neurologic disability, would exhibit notable cortical visual symptoms. However, there has been only one case report describing visual symptoms in detail in an encephalitis patient [6]. The present case took the severe disease course, and we inferred that it could relate to her characteristic visual symptoms.

There are many disorders of higher visual processing that result from damage to specific areas of the cerebral cortex which have a specific role in processing certain aspects (modalities) of vision. These can be grouped into those that affect the ventral, or “what?” pathway (e.g., object agnosia, cerebral achromatopsia, prosopagnosia, topographagnosia, and pure alexia), and those that affect the dorsal, or “where?” pathway (e.g., akinetopsia, simultagnosia, and optic ataxia) [7, 8]. The dorsal pathway may be subdivided further into two pathways: (1) the dorso-dorsal stream that extends from the primary visual cortex (V1) to the superior parietal lobule, which is supposed to be involved in implicit perception of an object's spatial position, motion, and form, and (2) the dorso-ventral stream that directs to the inferior parietal lobule being engaged in the conscious perception of an object's motion or spatial location [9].

Our patient showed a variety of higher visual symptoms which changed over recovery phase of the disease. In chronological order, she experienced cortical blindness, amblyopia, dyschromatopsia, relative difficulty in identifying stationary objects more than moving objects (static form agnosia), and prosopagnosia. On discharge, these visual symptoms recovered to the point where she had no complaints of disability in perceiving any visual stimuli. This implies that the patient may have unequally regained function of the aforementioned distinct visual areas in the time course of recovery. The areas involved in the dorsal pathway were likely improved initially, and those areas engaged in the ventral pathway were successively convalesced.

An intriguing observation in the present case was that, during the initial phase of her recovery, she demonstrated static form agnosia. Vania [10] previously reported a visually agnostic patient who suffered from bilateral occipital lesions, extending ventrally into the medial temporal areas. Similar to the present case, Vania's case showed impaired static form perception with preserved motion perception, including low-level motion perception (i.e., an ability to detect coherent motion when proportion of dots move coherently against a background of random dots), form-from-motion (i.e., an ability to detect kinetic boundaries defining shape), and biological motion perception (i.e., an ability to identify a point-light walker in the dark). Conversely, Blanke et al. [11] reported a case of impaired form-from-motion perception with preserved static form and low-level motion perception after damage to the ventral temporal lobe. These cases demonstrate a double dissociation suggesting that visual areas engaged in processing static form perception and form-from-motion perception rely upon functionally distinct regions. Consistent with this, functional MRI studies showed that the lateral occipital cortex and other ventral occipitotemporal cortices are activated when subjects were involved in detecting form-from-motion [12, 13], and there seemed to be little overlap in activation to static form stimuli and form-from motion stimuli within this region [14].

Because the patient suffered from encephalitis and the MRI revealed lesions with vague boundaries, detailed lesion localization in the present case was difficult to determine. However, it seems that two distinct visual areas (an area engaged in processing form-from-motion perception and an area involved in static form perception) recovered at different rates, with the former regaining function earlier.

Of note, the form-from motion task undertaken in our case is different from previous studies. In previous studies, form-from-motion tasks are performed as follows: a proportion of background dots in a random dot display move in one direction (or at a set velocity) comprising the background, and the remainder of dots comprising the foreground object (such as a letter) moving in another direction (or at a different velocity) [10–14]. The difference in the tasks performed in the present case and previous studies may prove difficult to directly compare results. However, the tasks in previous studies (and ours) both evaluated the subject's ability to detect kinetic boundaries. Moreover, the peculiar visual symptom demonstrated in the present case, i.e., “can see moving figures better than stationary ones,” suggests that these two visual perceptual abilities can be dissociable.

In conclusion, we presented a case of a variety of cortical visual symptoms in the recovery phase of anti-NMDA receptor encephalitis. The case further provides clinical evidence for functional segregation of static form and form-from-motion processing in the visual cortex.

## Figures and Tables

**Table 1:** Laboratory data.

WBC	16700/*μ*L	(3500–9700)	Influenza A	-	
RBC	490 × 10^4^/*μ*L	(376–516 × 10^4^)	Influenza B	-	
Plt	28.7 × 10^4^/*μ*L	(14.0–37.9 ×10^4^)	ANA	<40	(<79)
AST	12 IU/L	(8–38)	CEA/CLIA	1.1	(0.0–5.0)
ALT	10 IU/L	(4–44)	CA19–9	<=1.0	(0.0–37.0)
BUN	4.2 mg/dL	(8.0–20.0)	CA125	13.4	(0.0–35.0)
Cr	0.55 mg/dL	(0.47–0.79)	NSE/RIA	14.8	(0.1–16.3)
CPK	109 IU/L	(43–165)	CA602	10	(0–63)
UA	2.2 mg/dL	(2.7–7.0)	CSF		
Na	142 mEq/L	(135–145)	cell count	185/mm^3^	(0–15/mm^3^)
K	4.3 mEq/L	(3.5–5.0)	mono	168/mm^3^	
Cl	105 mEq/L	(98–108)	poly	13/mm^3^	
Glucose	105 mg/dL	(70–109)	protein	46 mg/dL	(15–45)
CRP	0.48	(0.00–0.30)	glucose	61 mg/dL	(50–75)
TSH	0.39*μ*IU/ml	(0.40–4.00)	HSV-PCR	-	
FT3	2.33 pg/ml	(1.71–3.71)			
FT4	1.19 ng/ml	(0.70–1.48)			
			Serum Anti-NMDAR antibodies	1:800	2 months after admission
				1:3200	4 months after admission

(), Normal range; Anti-NMDAR antibodies, Anti-*N*-methyl-D-aspartate receptor antibodies.

**Table 2:** Results of dot detection and figure recognition tasks.

	Stationary condition	Moving condition	Statistical values^a^
Dot detection	1/10 (correct/total)	8/10 (correct/total)	p < 0.001
Figure recognition	3/18 (correct/total)	7/18 (correct/total)	p = 0.06

^a^  Calculated by *χ*^2^ test using a 2 × 2 contingency table.
